# A Novel Tendon Injury Model, Induced by Collagenase Administration Combined with a Thermo-Responsive Hydrogel in Rats, Reproduces the Pathogenesis of Human Degenerative Tendinopathy

**DOI:** 10.3390/ijms25031868

**Published:** 2024-02-03

**Authors:** Laura Vidal, Maria Lopez-Garzon, Vanesa Venegas, Ingrid Vila, David Domínguez, Gil Rodas, Mario Marotta

**Affiliations:** 1 Leitat Technological Center, Carrer de la Innovació 2, 08225 Terrassa, Spain; 2 Bioengineering, Cell Therapy and Surgery in Congenital Malformations Laboratory, Vall d’Hebron Institut de Recerca (VHIR), Universitat Autònoma de Barcelona (UAB), 08035 Barcelona, Spain; 3 Medical Department of Futbol Club Barcelona (FIFA Medical Center of Excellence) and Barça Innovation, 08970 Sant Joan Despí, Spain; 4 Sports Medicine Unit, Hospital Clínic and Sant Joan de Déu, 08950 Barcelona, Spain; 5 Faculty of Medicine and Health Sciences, University of Barcelona, 08007 Barcelona, Spain

**Keywords:** animal model, collagenases, hydrogels, injections, patellar ligament, Pluronic, rats, tendinopathy, ultrasonography

## Abstract

Patellar tendinopathy is a common clinical problem, but its underlying pathophysiology remains poorly understood, primarily due to the absence of a representative experimental model. The most widely used method to generate such a model is collagenase injection, although this method possesses limitations. We developed an optimized rat model of patellar tendinopathy via the ultrasound-guided injection of collagenase mixed with a thermo-responsive Pluronic hydrogel into the patellar tendon of sixty male Wistar rats. All analyses were carried out at 3, 7, 14, 30, and 60 days post-injury. We confirmed that our rat model reproduced the pathophysiology observed in human patients through analyses of ultrasonography, histology, immunofluorescence, and biomechanical parameters. Tendons that were injured by the injection of the collagenase–Pluronic mixture exhibited a significant increase in the cross-sectional area (*p* < 0.01), a high degree of tissue disorganization and hypercellularity, significantly strong neovascularization (*p* < 0.01), important changes in the levels of types I and III collagen expression, and the organization and presence of intra-tendinous calcifications. Decreases in the maximum rupture force and stiffness were also observed. These results demonstrate that our model replicates the key features observed in human patellar tendinopathy. Collagenase is evenly distributed, as the Pluronic hydrogel prevents its leakage and thus, damage to surrounding tissues. Therefore, this model is valuable for testing new treatments for patellar tendinopathy.

## 1. Introduction

Musculoskeletal injuries can affect muscles, bones, and joint tissues, including tendons, ligaments, and cartilage. According to the World Health Organization (WHO), musculoskeletal injuries represent a major burden on individuals, as well as to health and social care systems, and they affect people of all ages worldwide [1]. Self-reported musculoskeletal diseases are highly prevalent (estimated to affect between 2% and 65% of the population). Moreover, their prevalence and effects are expected to increase with the aging of the global population [1,2]. Professional and recreational athletes are at a greater risk of injury due to their high levels of physical activity [3,4]. Among these pathologies, tendon injuries are prevalent and represent a significant clinical problem in sports-related medicine, accounting for 30% of general practice consultations for musculoskeletal pain [5,6].

Tendons are an essential part of the musculoskeletal system because of their structural role in connecting muscle and bone [5]. Injuries and abnormalities in the tendons are extremely common, often debilitating [6,7], and are typically diagnosed as sports injuries [8]. Injured tendons heal slowly and rarely recover the integrity of a healthy tendon, which results in clinical challenges and patient burden [4].

Damage to a tendon can result from an acute injury, such as a partial or complete tendon rupture, or chronic degenerative pathology (tendinopathy) [9]. The term tendinopathy describes the complex and multifaceted pathology of tendon injuries, which are characterized by pain, loss of function, and reduced exercise tolerance, resulting in a tendon that is less able to withstand repeated tensile loads [5,6,10]. It is identified by a disruption of the highly organized structure of collagen fibers, an increase in the non-collagenous extracellular matrix (ECM), hypercellularity, and neovascularization, among other conditions [5,11]. Tendinopathy affects more than 30 million people annually, impacting their health and quality of life, and it can even be career-ending for some athletes [12,13].

Based on clinical observations and statistical analyses, certain tendons, such as the patellar tendon, are more susceptible to injury [4]. Athletes participating in various sports exhibit an overall prevalence of 12% for patellar tendinopathy [14]. Patellar tendinopathy, or jumper’s knee, usually occurs in teenagers and young adults in the proximal part of the patellar tendon [7] and is associated with jumping sports, showing a higher prevalence in volleyball and basketball players [7,13,15,16,17,18].

Patellar tendinopathy is clinically diagnosed using imaging [13], with ultrasound (US) and magnetic resonance imaging (MRI) being the preferred clinical imaging techniques [15,19,20]. Power Doppler and color Doppler US imaging of the degenerated tendons typically reveal hypervascularity and neovascularization of the patellar tendon, which is associated with the pathogenesis [7,21,22]. Pathological features that are characteristic of tendinopathies, like alterations in the cross-sectional area (CSA), thickening of the tendon, and gray-scale ultrasonographic changes related to hypoechoic areas [23,24,25,26], are easily detected by means of US.

In addition, tendinopathy may be associated with calcific deposits inside the substance of a tendon, contributing to its symptoms [27]. The ossification of the tendon tissue within an injured tendon may increase the risk of tendon rupture and the incidence of post-operative complications and may result in a slow recovery time [28]. Such ossifications have been observed in imaging studies [20,29] and are thought to result from degenerative changes in the injured tendon. Achilles and patellar tendon ossifications are formed via a process resembling endochondral ossification, with bone formation and remodeling mediated by populations of osteoblasts and osteoclasts [28,30].

Among all the changes that occur during the development of tendinopathy, the role of inflammation has always been controversial, with various hypotheses proposed to describe the process, suggesting a significant cellular response to an injury [5,13,21,31]. However, several studies have suggested a potential role for immune cells in tendon healing [32] and in the evolution of tendinopathy [33,34,35]. Schubert et al. observed the presence of macrophages and T and B lymphocytes in Achilles tendinopathy samples [36]. The presence of inflammatory cytokines has also been associated with tendinopathy, as evidenced by the upregulation of IL-6 family members [37] and alterations in the TNF-α system [38]. However, the mechanisms underlying the persistence of inflammation, its resolution, and the role it may play in pathogenesis remain of interest in tendon diseases [39,40].

There is no standard treatment for tendinopathy, and available treatments are divided into surgical and non-surgical options. Non-surgical treatment is the primary option, and these therapies include exercise-based strategies, extracorporeal shockwave therapy, and injections of compounds such as platelet-rich plasma (PRP) or corticosteroids, among others [15,41,42]. The effect of treatment with percutaneous electrolysis, combined with exercise, in patients with patellar tendinopathy has recently been studied and may have an impact on improving pain and disability [43]. On the other hand, novel therapies that show potential for treating tendinopathy, such as scaffolds, stem cells, or gene therapy, are currently being developed [42].

To elucidate the pathogenesis of tendinopathy and to further develop novel therapeutic strategies for treating patients, the generation of in vitro and in vivo models represents an urgent need [5,44]. In vitro models are essential tools for studying the tendon microenvironment, cellular cross-talk, and the mechanisms of response to tendon mechanical loading [45,46]. They also play a crucial role in understanding the changes that occur in tendon cells due to pathology and are key to the development of potential treatments [47]. Explant cell cultures have been used to study the communication between the stromal collagen and the vascular compartments of tendons when temperature or oxygen levels are altered [48], as well as to study the development of the chondrogenic differentiation of tendon cells under pathological conditions [49]. More recently, 3D cultures have been developed through bioprinting to create tendon-on-chip models [50] and through controlled 3D architectures of electrospun microfibers to study the differences between pathological and healthy tendons [51]. In addition to the knowledge gained from in vitro models, using in vivo models is essential for understanding the dynamic interactions within living organisms, thereby providing a broader view that includes complex physiological responses and interactions. Although several in vivo models have been used to study tendon pathology [5], the lack of representative animal models closely mimicking human disease has hindered tendinopathy research [52]. Depending on the method used, the generated animal model presents specific characteristics of tendon pathology [53]. Collagenase injection is one of the most commonly used methodologies to generate tendon injury [54,55,56,57], since it reproduces many of the features seen in human tendinopathy, such as hypercellularity, loss of matrix organization, increased vascularity, and changes in the echogenic intensity of US tendon images [44,53].

Different types of enzymes can degrade the collagen matrix, but the most commonly used enzyme for generating tendinopathy models is collagenase type I, although there is no established consensus regarding the concentration and volume of collagenase to be injected [58]. De Cesar Netto et al. tested different doses and serial injections of collagenase for the development of an Achilles tendinopathy model in rabbits [59]. This model was based on the rat model developed by Perucca Orfei et al., in which they observed more visible changes in tendons treated with a higher dose of collagenase [55]. Similarly, Ghelfi et al. developed a porcine model of patellar tendinopathy and evaluated neovascularization and neovessel formation [60]. Similar models have also been used to evaluate molecular changes [61] and alterations in the biomechanical and US properties of injured tendons [62], increases in the expression of substance P and calcitonin gene-related peptide [63], and the presence of calcifications [64], which are associated with tendinopathy. On the other hand, when a collagenase liquid solution is injected inside a tendon, its distribution is usually not completely homogeneous, and a portion of it can spread outside the tendon, causing undesired damage to adjacent tissues. Acute inflammation and significant damage to the tissues surrounding a tendon have been described with the use of the liquid injection of enzymes that degenerate the ECM [65,66]. Other models have been developed to prevent the diffusion of these agents to unwanted areas and to promote more localized tendon injury generation through the use of kartogenin–alginate beads [67] or fibrinogen [57]. Therefore, using collagenase-containing thermo-responsive hydrogels could also solve this problem and allow for the localized and targeted administration of collagenase to produce an optimal tendon injury model.

Hydrogels are hydrophilic polymer networks that are able to retain large amounts of water or biological fluids; they can be either chemically stable or degradable, and they eventually disintegrate and dissolve [68]. Because of their composition, some types of hydrogels are thermo-responsive, such as Pluronic F127, which shows a fast gelation process at 37 °C and is a non-toxic, biodegradable, and FDA-approved hydrogel [69,70]. Using hydrogels to optimize the delivery of intra-tendinous injected collagenase could facilitate the development of novel and more accurate tendinopathy models to imitate human degenerative tendon pathology. Hydrogels have been widely used for tissue engineering applications [71,72,73,74], controlled release of therapeutic agents [75,76], cell delivery [77], and new treatment strategies in cancer [78,79].

This study presents a novel animal model of degenerative patellar tendinopathy that is induced via the US-guided injection of collagenase mixed with Pluronic hydrogel. In this study, the longitudinal evolution of collagenase–Pluronic-induced degenerative patellar tendinopathy was evaluated via histological and biomechanical testing to assess the degree of tendon degeneration or tendon healing [55,80,81]. This model may provide a valuable in vivo approach for studying tendinopathy and evaluating the efficacy of innovative regenerative therapies in future in vivo studies.

## 2. Results

### 2.1. Histology and Immunofluorescence Analysis of Patellar Tendon Injury

To evaluate the changes produced in the patellar tendon in the rat model, histological and immunohistochemical analyses were performed on both healthy (H) control and injured samples at each post-injury time point after the injection of Pluronic hydrogel (P) alone or a collagenase–Pluronic hydrogel (CP) combination into the patellar tendon. Collagenase mixed with Pluronic hydrogel was used to optimize and improve existing animal models that mimic tendon pathology [55,59,60,65,66].

**Figure 1:**
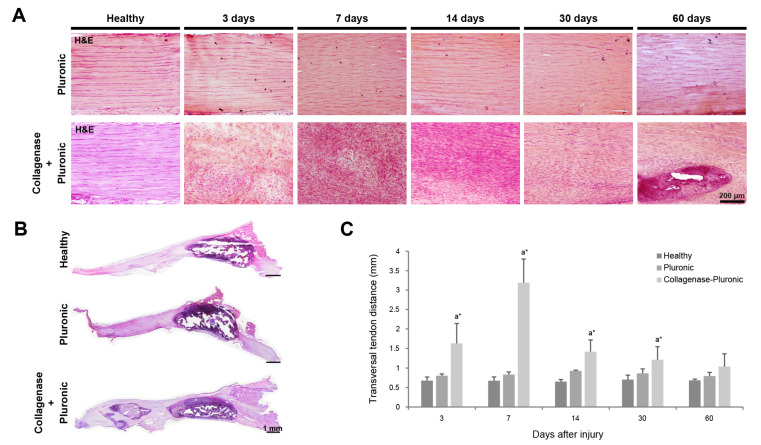
Histological analysis of rat patellar tendons. Tendon dimensions were analyzed using H&E-stained patellar tendon sections. Representative images show H&E-stained sections (**A**,**B**) of healthy, Pluronic hydrogel-treated, or collagenase–Pluronic hydrogel-treated patellar tendons. Measurement of the transversal dimensions (**C**) of rat patellar tendons after treatment at different points in time. Intergroup comparisons: ^a^ collagenase–Pluronic treated vs. healthy; * *p* < 0.01.

Through hematoxylin–eosin (H&E) staining, we analyzed the longitudinal evolution of the structural characteristics of the tendon tissue. We found intense tissue disorganization and hypercellularity in the tendons of the CP group at all time points (Figure 1A). For the longest post-injury time points, we also observed a tendency of nucleus and fiber realignment and the presence of intra-tendinous calcifications at 60 days post-injury. The administration of Pluronic hydrogel alone in the P group produced only a slight disorganization in the tendon structure and fiber alignment (Figure 1A). The size of the patellar tendons was also evaluated after the injections, and the transversal dimensions of the tendons (Figure 1B,C) were measured and quantified. The CP group showed a significant increase in tendon size, reaching a maximum transversal size (3.19 ± 0.61 mm) around 7 days post injury. Subsequently, the size of the tendons in the CP group gradually decreased until 60 days post-injection (1.04 ± 0.33 mm), at which point no significant size differences were observed compared to the H group’s tendons. The tendons of the P group did not show significant differences compared to the H group’s tendons throughout this longitudinal study.

The expression levels of structural (collagen types I and III), vascular (α-SMA and CD31), and inflammatory (CD68 and CD206) markers were determined by means of immunofluorescence analysis.

**Figure 2:**
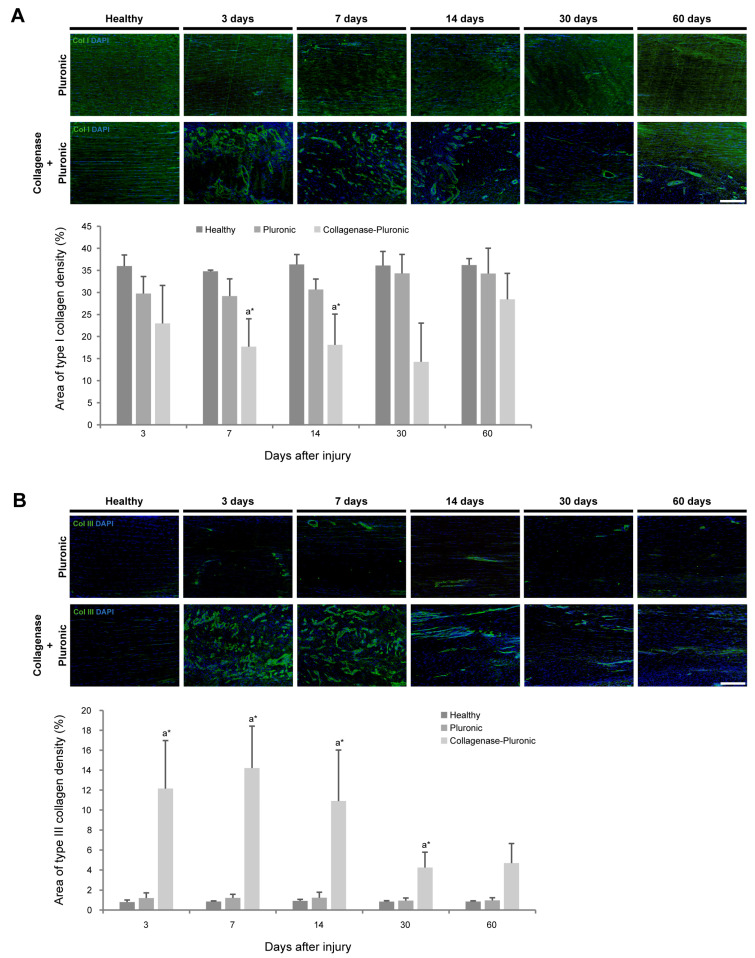
Immunohistochemical analysis of collagen types I and III in rat patellar tendons. Tendon structural characteristics were analyzed via immunofluorescence staining of patellar tendon sections. Representative images show photomicrographs (10×) for both collagen types I (**A**) and III (**B**) of healthy, Pluronic hydrogel-treated, or collagenase–Pluronic hydrogel-treated rat patellar tendons. The scale bar indicates 200 µm. Values of the area of expression (%) are presented. Intergroup comparisons: ^a^ collagenase–Pluronic treated vs. healthy; * *p* < 0.01.

Important changes in the organization of collagen types I and III were observed in the tendons of the CP group, which showed significant structural changes during the longitudinal evolution of the tendon injury. Collagenase administration provoked a prompt and substantial tissue disorganization in the CP group during the first weeks following the injection. However, their tendons recovered the structure and fibrillary organization from 30 days post-injury onwards, showing an improvement up to 60 days post-injury. In contrast, the administration of Pluronic hydrogel alone in the P group produced only minor structural changes in the patellar tendon. In particular, the analysis of type I collagen expression (Figure 2A) in the CP group showed a substantial decrease in collagen-I levels, reaching the highest decline (14.28 ± 8.76%) at 30 days post-injury. Interestingly, the collagen-I levels were almost recovered and reached similar levels to those observed in the H group at 60 days after injury. In contrast, the P group showed a slight non-significant decrease of around 4%.

On the other hand, the analysis of type III collagen expression levels (Figure 2B) showed the opposite phenomenon to that observed with type I collagen. Thus, a substantial increase in collagen-III levels (more than 10-fold) was promptly detected from 3 to 14 days post-injury, reaching its peak at 7 days after injury (14.22 ± 4.20%) and showing a progressive decline from 14 days to 30 and 60 days post-injury (4.68 ± 1.97%). Again, the tendons of the P group showed only slight changes, revealing a collagen-III expression pattern similar to that of the H group.

**Figure 3:**
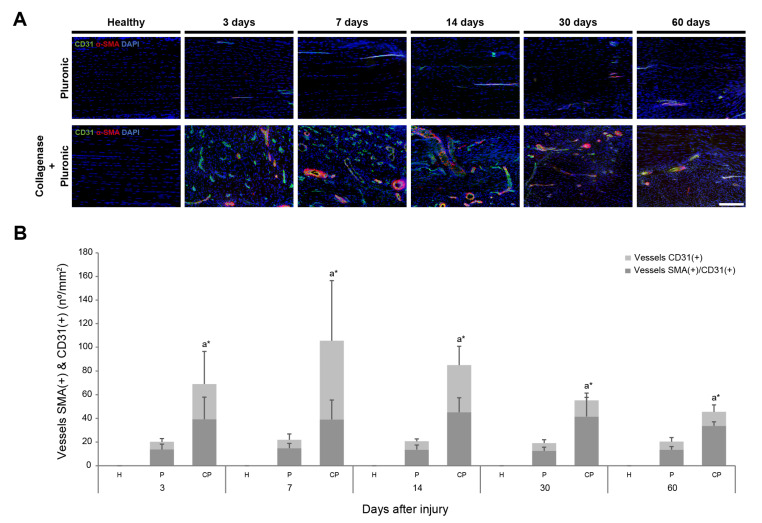
Neovascularization analysis of rat patellar tendons. The presence of vessels positive for angiogenic markers inside the rat patellar tendons was analyzed using immunofluorescence-stained patellar tendon sections. (**A**) Representative images show photomicrographs (10×) of vessels positive for α-SMA and CD31 markers inside healthy, Pluronic hydrogel-treated, or collagenase–Pluronic hydrogel-treated rat patellar tendons. The scale bar indicates 200 µm. (**B**) Quantification of vessels positive for α-SMA and CD31 (number of vessels/mm^2^). H: healthy; CP: collagenase–Pluronic hydrogel; and P: Pluronic hydrogel. Statistical results are shown for the total number of positive vessels. Intergroup comparisons: ^a^ collagenase–Pluronic treated vs. healthy; * *p* < 0.01.

The presence of vessels that were positive for the angiogenic markers CD31 (a marker for vascular endothelial cells of blood vessels [82,83]) and α-SMA (which stains the smooth muscle layer of blood vessels [84,85]), alone or co-expressed, was evaluated in the CP and P treatment groups. The patellar tendons in the CP group showed a prompt and strong increase during the first 2 weeks after collagenase administration, which then decreased at 30 and 60 days after injury. In the P group, only a few small vessels were observed (Figure 3A). An increase in α-SMA(+) and CD31(+) blood vessels was promptly observed on day 3 after injury in the CP group, reaching a maximum number of positive vessels at 7 days post-injury (105.46 ± 67 vessels/mm^2^) (Figure 3B). Between 14 and 60 days post-injury, there was a progressive decrease in α-SMA(+) and CD31(+) blood vessels. Despite this, a significant number of α-SMA(+) and CD31(+) blood vessels remained detectable even 2 months after collagenase administration in the CP group (45.56 ± 8.87 vessels/mm^2^). In contrast, the injection of Pluronic hydrogel alone in the P group resulted in a minor presence of α-SMA(+) and CD31(+) blood vessels, which remained relatively constant over the subsequent 2 months.

**Figure 4:**
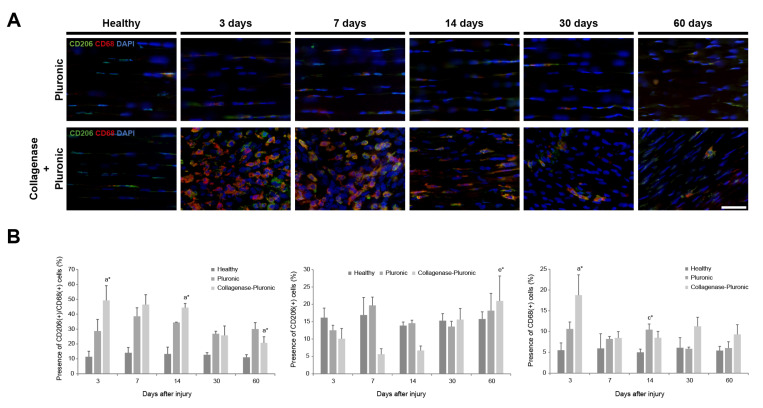
Analysis of inflammation-related cells in rat patellar tendons. The inflammatory response in the injured tendons was also evaluated based on the presence of cells expressing the CD68 and CD206 inflammatory markers using immunofluorescence-stained patellar tendon sections. (**A**) Representative images show photomicrographs (40×) of cells positive for CD68 and CD206 markers in healthy, Pluronic hydrogel-treated, or collagenase–Pluronic hydrogel-treated rat patellar tendons. The scale bar indicates 50 µm. (**B**) Quantification (%) of cells positive for CD206(+) or CD68(+) alone and cells double-positive for CD206(+)/CD68(+). Intergroup comparisons: ^a^ collagenase–Pluronic treated vs. healthy, ^c^ Pluronic treated vs. healthy; intragroup comparisons: ^e^ compared to 7 days post-injury; * *p* < 0.01.

The CD68 and CD206 inflammatory markers were used to evaluate the inflammatory response in the injured tendons. CD68, a marker associated with monocytes/macrophages [86,87], and CD206, a well-known marker for identifying the M2 macrophage subtype [88], were analyzed in the H group and the CP and P treatment groups (Figure 4A). During the initial 3–14 days post-injury, the tendons in the CP group showed a higher number of CD68-positive cells (11.90 ± 5.94%) than CD206-positive cells (7.45 ± 3.16%). However, this pattern reversed 30 days after injury, with a higher proportion of CD206-positive cells (18.28 ± 3.80%) than CD68-positive cells (10.27 ± 1.38%). A similar trend was observed in the P group, where CD206-positive cells outnumbered CD68-positive cells. In the H group, the tendons consistently expressed both markers over time, with a higher proportion of cells positive for CD206 than for CD68 (Figure 4B). The presence of CD68(+)/CD206(+) double-positive cells (Figure 4B) remained relatively constant over time in the H and P groups, with higher values in the P group. In the CP group, there was a higher presence of these cells during the initial 3–14 days after injury (46.74 ± 2.41%). However, this proportion decreased by half at 30 to 60 days after injury (23.20 ± 3.53%).

### 2.2. Ultrasound Clinical Imaging Analysis of the Longitudinal Evolution of Patellar Tendon Injury

**Figure 5:**
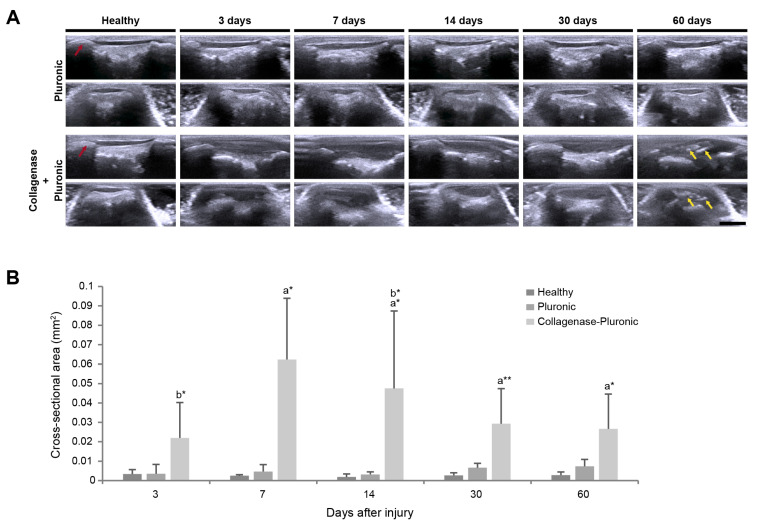
Longitudinal evolution of rat patellar tendons via clinical US imaging. (**A**) Representative US images of patellar tendons (*N* = 6 for each time point) in both longitudinal (upper panels) and transverse (lower panels) views. The scale bar indicates 3 mm. The red arrows indicate the patella (kneecap bone), and the yellow arrows indicate intra-tendinous calcifications. (**B**) Measurement of the cross-sectional area (mm^2^) of rat patellar tendons. Intergroup comparisons: ^a^ collagenase–Pluronic treated vs. healthy, ^b^ collagenase–Pluronic treated vs. Pluronic treated; * *p* < 0.01, ** *p* < 0.001.

Through clinical US imaging, we monitored the longitudinal evolution of the rat patellar tendons and assessed their evolution at each time point. As shown in Figure 5, US was used to confirm the accurate delivery of the Pluronic hydrogel or the collagenase–Pluronic mixture inside the rat patellar tendons. Additionally, it was used to evaluate the anatomical and structural changes in the treated tendons, considering echogenicity and size variations.

In contrast to healthy human tendons that appear as echogenic fibrillar structures [89], healthy rat patellar tendons of the H group showed an anechoic signal on clinical US imaging. Injured rat patellar tendons showed an increase in echogenicity, indicating that the injured tendons of the CP group were hyperechogenic compared to the tendons of the H group. After the injection of the collagenase–Pluronic mixture (Figure 5A), differences in tendon appearance were observed in the CP group in both the longitudinal and transversal axes, which were accompanied by signs of inflammation. Notably, at 60 days post-injury, we observed the presence of intra-tendinous calcifications in the tendons of the CP group (marked with yellow arrows, Figure 5A). According to the US images, these calcifications within the tendons appeared as hyperechoic spots with hypoechoic shadows underneath, a characteristic feature seen in bone detection images. Contrastingly, the patellar tendons in the P group exhibited a similar appearance to those in the H group, with no calcifications detected within the tendon during the US evaluation.

The cross-sectional area (CSA) of the patellar tendons was also measured in the different treatment groups using the US images (Figure 5B). CSA values were longitudinally evaluated throughout the whole study and were compared between the baseline and the final time point for each animal. A significant increase in CSA values was promptly observed in all injured tendons in the CP group, showing a maximum increase (0.062 ± 0.032 mm^2^) when compared to the H group’s tendons (0.002 ± 0.001 mm^2^) at 7 days post-injury. At 14 days post-injury, the CSA values of the CP group started to progressively decrease, but still showed an almost 10-fold increase at 60 days after injury (0.027 ± 0.018 mm^2^). In contrast, the patellar tendons of the P group showed a slight increase in the CSA, reaching a maximum 2.5-fold increase compared to tendons of the H group.

**Figure 6:**
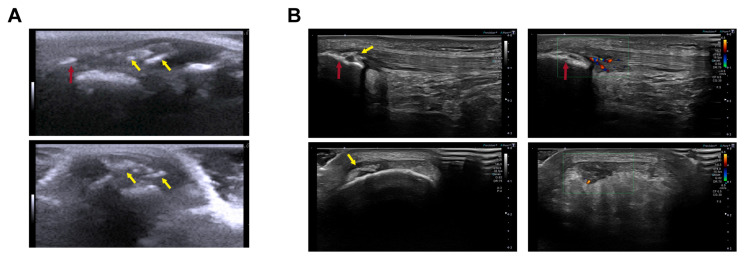
US in vivo imaging of tendon intra-tendinous calcifications for comparison between the collagenase–Pluronic treated rat patellar tendinopathy model and the images from human professional athletes. The red arrows indicate the patella, and the yellow arrows indicate intra-tendinous calcifications. The green rectangle indicates the area where Doppler US was analyzed. Longitudinal views (upper panels) and transversal views (lower panels) of the patellar tendons are shown. (**A**) Representative US images of the rat tendinopathy model at 60 days after injury, when intra-tendinous calcifications and substantial tendon swelling can be observed. (**B**) Representative US images of human patellar tendinopathy (images from a 23-year-old male professional basketball player), showing the presence of calcifications in the proximal region of the patellar tendon ((**B**), left panels), and Doppler US imaging indicating neovascularization with infiltrating blood vessels ((**B**), right panels).

Additionally, we evaluated similarities between our rat model of patellar tendinopathy induced via the injection of the collagenase–Pluronic mixture and observations in human athletes. The evaluation was performed by comparing US images obtained from the rat model with those acquired from professional basketball players. As shown in Figure 6, we demonstrated that our patellar tendinopathy model in rats faithfully reproduces the characteristics observed in athletes during the US evaluation. In our rat model, we observed the presence of inflammation, along with a significant increase in the size of the tendon and the development of intra-tendinous calcifications 2 months after injury (Figure 6A). The echogenic signs detected in these rats closely resembled those observed in the patellar tendons of professional basketball players. These signs included an increase in tendon size, neovascularization with blood vessel infiltration, and calcifications in the proximal insertional region or within the patellar tendon itself (Figure 6B).

### 2.3. Biomechanical Analysis of Patellar Tendon Injury

**Figure 7:**
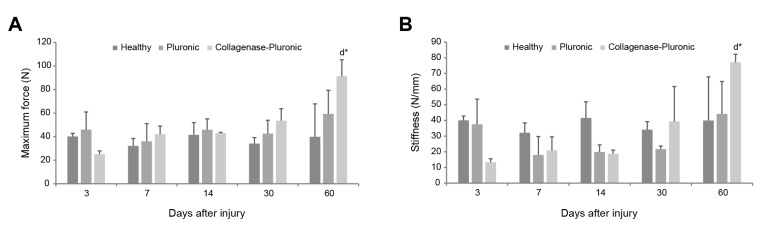
Biomechanical properties of rat patellar tendons. Evaluation of changes in the tendon biomechanics via (**A**) measurement of the maximum tendon rupture force (N) and (**B**) measurement of tendon stiffness (N/mm). Intragroup comparisons: ^d^ compared to results 3 days post-injury; * *p* < 0.01.

The biomechanical properties of the injured tendons were evaluated using the rat model. On the one hand, we assessed the maximum rupture force (load-to-failure) values of the patellar tendons (Figure 7A), revealing important changes in tendon biomechanics. There was a notable decrease in tendon force observed in the CP group at 3 days after injury (53.83 ± 9.70 N and 25.12 ± 2.79 N in the H and CP groups, respectively). The tendons of the H group exhibited a stronger rupture force compared to those of the P and CP groups. However, at 60 days post-injury, we observed the opposite effect, and the tendons of the CP group presented a significantly higher maximum rupture force (58.98 ± 25.20 N and 91.15 ± 14.13 N in the H and CP groups, respectively).

On the other hand, we measured the stiffness of the patellar tendons (Figure 7B), defined as the force required to deform the tissue. The stiffness showed a similar pattern to the maximum rupture force measurement, with the H group showing higher stiffness values than the P and CP groups. However, at 60 days after injury, the CP group showed higher stiffness values than did the H group. The patellar tendons of the CP group presented stiffness values that were lower than those measured for the tendons of the H and P groups at 3 days after injury (40.03 ± 2.73 N/mm, 48.87 ± 21.43 N/mm, and 31.19 ± 18.70 N/mm in the H, P, and CP groups, respectively). Over time, the stiffness values increased, reaching the highest values among the different groups at 60 days post-injury (39.94 ± 27.84 N/mm, 44.16 ± 20.71 N/mm, and 77.06 ± 5.19 N/mm in the H, P, and CP groups, respectively).

## 3. Discussion

In this study, we present a novel animal model of degenerative patellar tendinopathy induced in rats via intra-tendinous collagenase injection guided by US, using a Pluronic thermo-responsive hydrogel as a vehicle for collagenase administration. While previous studies have reported the use of gels to optimize the delivery of collagenolytic enzymes, such as the use of a fibrin gel to induce tendinopathy via collagenase injection in an equine model [57], or the use of a thermo-responsive chitosan–gelatin–glycerol–phosphate hydrogel as a collagenase carrier for early fibrovascular tissue regeneration [90], the use of thermo-responsive gels to optimize a collagenase-induced tendinopathy model has not been previously reported. In the present work, we introduced, for the first time, a combination of collagenase and a thermo-responsive hydrogel, specifically Pluronic F127, to generate a model of patellar tendinopathy. Type I collagenase was selected as the collagenolytic agent [91] for inducing the degeneration of the tendon tissue to produce tendon injury, a method that was previously employed in established models replicating this pathology [54,55,56,58,59,60,61]. Pluronic F127 hydrogel was chosen for its documented properties as a thermo-responsive, non-toxic, biodegradable, and FDA-approved hydrogel [69,70].

Numerous animal models of tendinopathy have been induced through the use of the intra-tendinous injection of collagenolytic enzymes, with the majority involving the direct injection of collagenase in a liquid form [55,59,60,61,65,66]. One common limitation of this model is the heterogeneous distribution of these enzymes, leading to irregular and inconsistent injuries or undesired damage to adjacent tissues. In existing animal models of tendinopathy, significant damage, with a subsequent inflammatory response in the surrounding tissues, has been described when ECM-degrading enzymes are injected into the targeted tendon [65,66]. To overcome this issue, we mixed collagenase with Pluronic hydrogel to ensure a uniform distribution of collagenase throughout the patellar tendon. The collagenase–Pluronic solution was injected homogeneously into the patellar tendon, and it rapidly gelled as the temperature increased to the animal’s body temperature.

The collagenase/Pluronic mixture ratio used was 1:4, based on studies conducted by Meng et al. [92]. This ratio was found to be optimal at preventing the spread of collagenase and unwanted damage to adjacent tissues. The great potential of hydrogels in preventing the diffusion of ECM-degrading enzymes when developing tendinopathy models has been previously reported. Yuan et al. created localized tendinopathic lesions by injecting kartogenin–alginate beads [67], and Watts et al. used a fibrinogen gel as a collagenase carrier in their tendinopathy model [57].

In the present work, we evaluated the precision and reproducibility of collagenase–Pluronic hydrogel administration and the longitudinal evolution of tendon injury via clinical US imaging. The changes produced during the degenerative injury and healing process were determined using histological and immunofluorescence analyses. Additionally, we evaluated the biomechanical and functional properties of the treated patellar tendons by performing load-to-rupture tests to thoroughly characterize the model. H&E staining was performed on the tendon specimens, while specific markers, including collagen types I and III, α-SMA/CD31, and CD68/CD206, were evaluated by means of immunofluorescence analysis. These assessments aimed to identify the presence of structural, vascular, and inflammatory changes. The effects of the degenerative injury were further confirmed via in vivo clinical imaging using US and biomechanical load-to-rupture analysis. Our analyses collectively demonstrated that our model follows the same degenerative processes observed in similar animal models [64,93,94] and in human patients [95].

The obtained histological data confirmed that our rat model of patellar tendinopathy showed signs of tissue disorganization, hypercellularity, and intra-tendinous calcifications at 60 days post-injury, as well as an increase in tendon size, as observed in other models established to reproduce this disease [64,96]. We further demonstrated that our model showed the structural and vascular changes typically observed in this type of injury. The analyses of collagen types I and III showed substantial variations. In our rat model, type III collagen was upregulated, while type I collagen was downregulated, a phenomenon that has been widely reported to occur naturally in tendinopathies [57,96,97,98]. In contrast to healthy tendons, tendinopathic rat patellar tendons showed important changes in collagen expression and organization, resulting in a loss of the classical hierarchical structure. Notably, type I collagen expression showed a significant decrease in tendinopathic patellar tendons, indicating an evident degradation of the fibrillary tendon structure, as has been observed in other studies on tendinopathy [54,99,100,101]. A major disorganization of the tissue was observed, resulting in a loss of the hierarchical collagen I structure. This structure is mainly responsible for the tendon’s mechanical characteristics, and its loss could lead to a reduced ability to withstand high loads. Additionally, type III collagen expression showed a prompt and strong increase (a greater than 12-fold increase) in the first 14 days, which is a common molecular change observed in tendinopathy [11]. Previous studies have reported that abnormal amounts of type III collagen are associated with wound healing, since type III collagen is produced in the initial phases of tendon damage to rapidly “patch” the injured area [2,6].

The prompt and strong increase in α-SMA and CD31 vascular markers demonstrated the appearance of strong intra-tendinous neovascularization, which is also associated with the natural process of tendon repair [95,102]. We observed a contrast between avascular healthy patellar tendons and the increased neovascularization observed in injured tendons. As the degenerative injury progressed and the patellar tendon started to heal, the number of α-SMA(+) and CD31(+) blood vessels progressively decreased. These findings are consistent with power Doppler US imaging data obtained from athletes who suffer from tendinopathy, which also show increased tendon vascularity [21,25]. Some in vivo studies have associated this neovascularization with pain, possibly because of the generation of new nerves following the formation of new blood vessels [52]. Our model could be used to study new treatment options to reduce this pain and improve current treatment techniques, such as the injection of sclerosing agents to inhibit vessel formation [15,103,104], corticosteroid injection, or PRP therapies [52]. Importantly, the histological analysis of the injured tendons showed the presence of intra-tendinous calcifications in the rat model at 60 days after injury. Intra-tendinous calcifications are commonly detected in human tendinopathy [7,15,21] and can be considered one of the problems arising from a degenerative pathology that should be resolved in human clinics. In this sense, our model of degenerative tendinopathy in rats also provides an excellent model to study and treat intra-tendinous calcifications that can cause severe problems and morbidity in patients with tendinopathy.

The role of inflammation in tendinopathy is not clearly established and is still a matter of debate. Several studies, both in human and animal models, have suggested a potential role of immune cells in the tendon repair process [5,32,105]. The existence of resident macrophages and the presence of other immune cells and markers have been described in different tissues, including tendons. These cells play specific roles, such as maintaining tendon homeostasis, and could also be involved in the process of the repair and regeneration of tendon damage, as well as in resolving tendon inflammation [106,107]. More recently, tendon-resident cells expressing immune cell markers have been identified and described as “tenophages”, or macrophage-like tendon-resident cells [108]. Thus, we evaluated the presence of the inflammation-related markers CD68 (a monocyte/macrophage marker) and CD206 (an M2 subtype macrophage marker). A prompt and strong increase in the expression levels of CD68 and CD206 was detected in injured tendons, indicating the presence of macrophages or macrophage-like cells during the first days after injury and throughout the development of patellar tendinopathy. Macrophages play an essential role in modulating the inflammatory processes associated with the pathogenesis and resolution of tissue injury and inflammation [108]. The presence of macrophages in both the control and tendinopathic tendons has also been observed in other studies [109]. We observed some inflammatory cells positive for CD68, especially during the first days after injury, that are found in specimens obtained from acutely ruptured tendons [109] and non-ruptured chronic tendinopathic tendons [110]. The presence of macrophages in an injured tendon has been reported to accelerate the healing process by increasing cell proliferation and ECM generation. However, the presence of macrophages may decrease the mechanical properties of the tendon [111]. We also observed a significant increase in cells positive for the CD206 marker, especially in the first 60 days after injury. This finding corroborates previously reported data regarding the presence of M1 macrophages throughout the tendon injury evolution and the accumulation of M2 macrophages at sites of ECM reorganization, becoming the predominant phenotype at 4 weeks after injury [112]. The M2 macrophage subtype increases its concentration as the healing process advances and seems to play a role in the termination of inflammation by stimulating new tissue deposition and cell proliferation within the injured tendon [113]. On the other hand, other studies have reported that the accumulation of M1 macrophages in the first days after injury did not induce changes in the expression of M2 macrophages during the evolution of tendon injury [114]. All these results indicate that further studies investigating the relationship between inflammation and tendinopathy are still needed.

Most sports-related injuries in humans are commonly assessed using non-invasive imaging techniques such as US, despite the operator-dependent nature and limited soft-tissue contrast inherent in these methods [15,26]. In this study, we evaluated the progression of patellar tendinopathy in our rat model using clinical US imaging. Remarkably, we observed a substantial increase in the tendon CSA dimensions in the CP group due to the degenerative injury. The increase in CSA suggests severe inflammation and swelling of the tendinopathic tendon [6], a finding supported by similar observations in studies of tendinopathy in human athletes [24]. Additionally, we identified intra-tendinous calcifications at 60 days post-injury, confirming the histological findings obtained using our rat model. These calcifications were clearly visible in the tendon body and could occupy up to 5% of the tendon area. Such calcifications are a common sonographic finding, as well as a clinical manifestation associated with human tendinopathy [7,15,21]. The origin of intra-tendinous calcifications could be attributed to the aberrant differentiation of resident cells in an injured tendon or the presence of other cell types that migrate to the injured tendon to participate in the regenerative process. These cells could be influenced by their environment, and a combination of cytokines in the injured area could induce aberrant cell differentiation, causing cells to commit to the path of osteogenesis, resulting in the subsequent formation of calcifications.

This idea is supported by the fact that the aberrant cell differentiation of resident tendon cells, such as tendon stem cells (TSCs), into an osteoblastic lineage with a compromised capacity for tendon healing could be responsible for calcifications within the tendon tissue [5,21]. In particular, the capacity of TSCs for osteogenic differentiation has already been reported to be influenced by the signaling received by these cells and the activation of specific signaling pathways, such as ERK1/2 [115] and the Hedgehog signaling pathway [116], or the effect of reactive oxygen species [117]. These data support the potential role of tendon resident cells in generating intra-tendinous calcifications due to abnormal cell differentiation. It has also been observed that BMP-2-mediated effects on human TSCs may contribute to the formation of calcified tissues in tendinopathic tendons [118]. Moreover, this erroneous deposition of the extracellular matrix and bone tissue deposits could be responsible for weakening the tendon, resulting in failed healing and activity-related tendon pain [119].

To delve into the impact of tendon degenerative injuries, we also analyzed the biomechanical properties of the patellar tendon after an injury. The biomechanical analysis results showed a prompt decrease in the maximum rupture force in the CP group at 3 days post-injury, after which the biomechanics of the patellar tendons showed a progressive improvement until reaching values comparable to those of the H group. The decreased resistance to tendon rupture can be attributed to the disorganization of the tendon fibrillar structure and the appearance of non-native tendon tissue. As has been described in human tendinopathy, the formation of scar tissue instead of fibrillar and well-organized native tendon tissue can reduce tensile strength by up to one-third that of intact tendons [5]. In addition, the rapid appearance of many type III collagen fibrils, which may be smaller and less resistant than those formed by type I collagen, could also contribute to a rapid decrease in mechanical properties [53]. Interestingly, at 60 days post-injury, the tendons of the CP group showed an increased resistance to rupture by mechanical stretching, along with stiffness values higher than those of the H group. These findings are corroborated by previous animal studies reporting similar data in regards to increased maximum rupture force and stiffness over time in collagenase-injected rat Achilles tendons [62], as well as by several human studies in which the influence of tendinopathy has already been associated with changes in elastic properties and higher patellar tendon stiffness [24,120], thereby affecting the biomechanical properties of the injured tendon during the evolution of tendinopathy.

The results of our study have broader implications for the field of tendon pathology research by providing a more comprehensive understanding of disease progression and prospective targets for intervention, such as the development of potential therapies for the treatment of calcific deposits within an injured tendon [121]. In addition, the successful development of this model opens up new avenues for translational research, with promising clinical applications. Pluronic hydrogels have been tested in several pre-clinical studies, and some clinical trials are currently testing their use in various therapies [122]. The model we developed has the capacity to advance therapeutic strategies and contribute to the development of targeted treatments for patellar tendinopathy and potentially other related tendon disorders [42], including the development of novel stem cell-based [12,54,80], drug delivery-based [123,124], or gene-based therapies [125,126].

We acknowledge several limitations of our study. First, this study included a limited sample size, which may affect the generalizability of our findings and have implications regarding the robustness of the statistical analysis. In addition, the rat model developed in our study may possess limited translatability to humans. Conducting similar studies in larger animal models may increase the relevance and applicability of our findings to human conditions. In addition, given the lack of consensus regarding the use of collagenase concentrations to induce tendinopathy models, different concentrations may have different effects, which were not examined in our study.

## 4. Materials and Methods

### 4.1. Animals

A total of 60 adult (3-month-old) male Wistar rats (Envigo Laboratories, Indianapolis, IN, USA), with a mean body weight of 389.45 ± 33.93 g, were used in this study. The rats were housed at 22–24 °C on a 12 h light/dark cycle. Tap water and food were offered ad libitum during the experiment. All procedures were performed following Spanish (Real Decreto 53/2013) and European (2010/63/UE) regulations and were approved by the Departament d’Agricultura, Ramaderia, Pesca, Alimentació i Medi Natural of the Catalan Government, under procedure number 52/17.

The sample size was calculated according to Mead’s resource equation (E = N-T, 10 < E < 20), as carried out in other animal models of collagenase-induced tendinopathy [55,60]. Considering that for each time point (3-, 7-, 14-, 30-, and 60-day post-injection), half of the tendons were used for functional load-to-failure force analysis and the other half for histological analysis, we considered two groups for analysis per time point (T = 2 analysis × 5 time-points = 10). Our calculations for each analysis were based on the use of 3 animals (N = T × 3 = 30), so the sample size was calculated as follows: E = (30 − 1) − (10 − 1) = 20. Thus, 30 animals were used for each group, including the collagenase–Pluronic treated (CP) and the Pluronic treated (P) groups. The animals were randomly divided into 5 treated groups (N = 6 per group/time) following time points at which euthanasia was carried out—3, 7, 14, 30, and 60 days post-injection. Prior to euthanasia, half of the animals were randomly assigned to the functional load-to-failure force analysis group (N = 3) and the other half to the histological analysis group (N = 3). In the functional load-to-failure force analysis, the tendon tissue was destroyed and became non-viable for further histological analysis, thus requiring the allocation of half of the animals to each type of analysis. In all animals, the contralateral tendon was left untreated and was used as a healthy (H) control.

### 4.2. Generation of Patellar Tendinopathy Model in Rats

The animals were anesthetized and maintained in this state (5% and 2%, respectively) via the inhalation of isoflurane (Baxter International Inc., Deerfield, IL, USA), and they were placed in a supine position, with the right knee joint facing up prior to the surgical procedures. The knee joint was shaved and sterilized with 70% ethanol before injection.

The rats were injected, in the right patellar tendon, with a mixture of Pluronic F127 hydrogel (BASF, Ludwigshafen, Germany) and collagenase type IA (collagenase from Clostridium histolyticum, Sigma-Aldrich, St. Louis, MO, USA) or with Pluronic F127 hydrogel alone. Collagenase is an enzyme that breaks the peptide bonds of collagen [55,91], destroying the tendon’s collagen fibers and generating pathological effects. To achieve a homogeneous distribution of collagenase, we used a hydrogel with thermo-responsive properties that allows a homogeneous and controlled release of collagenase [90,92,127,128]. Specifically, Pluronic F127 is a biodegradable, non-toxic, and FDA-approved hydrogel with thermo-responsive properties [69,70], making it a good candidate for use in our model. Each injection contained 20 μL of the mixture of collagenase (3 mg/mL) + Pluronic hydrogel in a proportion of 1:4 (final collagenase solution of 0.3%), or 20 μL of Pluronic hydrogel mixed with a saline solution, which was also in a proportion of 1:4 [55,92]. The collagenase was previously dissolved in a saline solution, filtered for sterilization using a 0.22 μm filter (Merck KGaA, Darmstadt, Germany), and mixed with the hydrogel under sterile conditions. The collagenase + Pluronic hydrogel and Pluronic hydrogel solutions were maintained at cold temperatures (2–8 °C) until the moment of injection to avoid the gelation of the hydrogel.

The sterile collagenase + Pluronic hydrogel or Pluronic hydrogel solutions were injected into the rat patellar tendons using a 30 G needle (BD Microlance™, Becton Dickinson, Franklin Lakes, NJ, USA). The intervention was performed under US guidance by inserting the needle into the proximal part of the tendon until it reached the deep fibers, and then 20 μL of the solution was released (Supplementary Materials, Video S1). By performing the injections under US guidance, we were able to employ a minimally invasive technique to avoid additional harm to the animals. This approach prioritized animal welfare and ensured precise and accurate injection to the desired location within the patellar tendon. After the surgical procedure, post-surgical analgesia (buprenorphine at 0.01 mg/kg) was administered subcutaneously to all animals. Careful monitoring was implemented to ensure the welfare of the animals after the induction of the degenerative injury, and regular examinations showed no signs of pain or morbidity after injection.

### 4.3. Ultrasound Clinical Imaging Analysis of the Longitudinal Evolution of Patellar Tendon Injury

For the US imaging analysis, animal anesthesia was induced and maintained via the inhalation of isoflurane (5% and 2%, respectively). All US procedures were performed using a portable MyLab ONE US device (Esaote, Genoa, Italy) and an SL3116 22 MHz linear transducer (Esaote, Genoa, Italy), with a frequency range of 8 to 22 MHz.

The images obtained from the patellar tendons were captured from both longitudinal and transversal views. The evolution of tendon pathology was longitudinally monitored via US imaging. The cross-sectional area of the patellar tendons was measured after marking the total transverse area of the tendon using Image J software (version 1.52, U. S. National Institutes of Health, Bethesda, MD, USA), based on a calibrated pixel ratio to the actual size (mm) of the area.

### 4.4. Histology and Immunofluorescence Analysis of Patellar Tendon Injury

Immediately after euthanasia, half of the animals (N = 3) from both groups (CP and P groups) were randomly selected at each time point for the histological analysis. The patellar tendons from both legs were extracted from the patella to the tibial insertion, including Hoffa’s fat pad. Then, the tendons were immediately placed in disposable base molds and covered with OCT^®^ Tissue Freezing Medium (Leica Biosystems, Nußloch, Germany). The specimens were immediately frozen in liquid nitrogen and stored at −80 °C until use. The frozen patellar tendon samples were longitudinally sectioned (10 μm thick) using a cryotome (Leica Microsystems, Wetzlar, Germany) at a temperature of below −20 °C and mounted on Polysine^TM^ glass slides (VWR, Radnor, PA, USA). Consecutive frozen sections were stored at −20 °C and used for subsequent histological and immunofluorescence analyses.

For the histological analysis, cryosectioned patellar tendons were stained with hematoxylin and eosin (2 min of hematoxylin and 1 min of eosin), dehydrated with graded ethanol solutions (ethanol 50%, ethanol 70% twice, ethanol 90% twice, and ethanol 100% twice, for 1 min each), cleared in xylene (5 s), and mounted with DPX mounting medium (Sigma-Aldrich, St. Louis, MO, USA) and a coverslip (VWR, Radnor, PA, USA). Harris’ hematoxylin solution was purchased from QCA (Química Clínica Aplicada S.A, Tarragona, Spain ), and the eosin solution was prepared by dissolving 0.5 g of Eosin Yellowish (Panreac Química S.L.U, Castellar del Vallès, Spain) in 100 mL of distilled water with 200 μL of glacial acetic acid (VWR, Radnor, PA, USA). Ethanol absolute and xylene were obtained from Panreac (Panreac Química S.L.U, Castellar del Vallès, Spain). The histological samples were evaluated using a BX61 microscope (Olympus, Tokyo, Japan), a DP72 camera (Olympus, Tokyo, Japan), and CellSens^®^ Digital Imaging software (version 1.9, Olympus, Tokyo, Japan). The images were measured using Image J software (version 1.52, U. S. National Institutes of Health, Bethesda, MD, USA) to determine the transversal dimensions of the tendon (mm).

For the immunofluorescence analysis, cryosectioned patellar tendons were fixed in cold (−20 °C) acetone (Química Clínica Aplicada S.A, Tarragona, Spain) for 10 min, air-dried for 5 min, and blocked in phosphate-buffered saline (PBS; VWR, Radnor, PA, USA) 1× containing 3% bovine serum albumin (BSA; Biowest, Nuaillé, France) for 10 min at room temperature. Then, the tendon sections were incubated overnight with primary antibodies diluted 1:100 in PBS 1X + 3% BSA in a dark, humid chamber at 4 °C. The primary antibodies used were against rabbit anti-collagen I (ab34710, Abcam, Cambridge, UK), rabbit anti-collagen III (ab7778, Abcam, Cambridge, UK), rabbit anti-α-smooth muscle actin, α-SMA (ab5694, Abcam, Cambridge, UK), mouse anti-rat CD31 (550300, BD-Pharmingen, Becton Dickinson, Franklin Lakes, NJ, USA), mouse anti-rat CD68 (MCA341R, Bio-Rad, Hercules, CA, USA), and rabbit anti-CD206 (ab64693, Abcam, Cambridge, UK). The samples were washed 5 times each in PBS 1X and then incubated with the secondary antibodies Alexa Fluor^®^ 488 donkey anti-rabbit (A21206, Life Technologies, Carlsbad, CA, USA) or Alexa Fluor^®^ 568 donkey anti-mouse (A10037, Life Technologies, Carlsbad, CA, USA), diluted to 1:1000 in PBS 1X + 3% BSA, in a dark, humid chamber for 1 h at room temperature. Finally, the samples were washed 5 times with PBS 1X and mounted using Fluoromount-G^TM^ with DAPI (Invitrogen, Waltham, MA, USA). Fluorescence was then evaluated using a BX61 microscope (Olympus, Tokyo, Japan) equipped with a DP72 camera (Olympus, Tokyo, Japan) and CellSens^®^ Digital Imaging software (version 1.9, Olympus, Tokyo, Japan). The images were measured using Fiji software (version 2.14.0, U. S. National Institutes of Health, Bethesda, MD, USA) to evaluate the density area of collagen I and collagen III and to determine the presence of α-SMA and CD31-positive blood vessels or CD68- and CD206-positive inflammatory cells in the tissue samples.

### 4.5. Biomechanical Analysis of Patellar Tendon Injury

For biomechanical studies, the patellar tendons of both the injured and healthy contralateral legs were harvested immediately after euthanasia from half (N = 3) of the animals of each group (Groups CP and P) at every time point. The tendon specimens were immediately used for maximum rupture force (load-to-failure) analysis by clamping them vertically into the grips of a Z010 universal testing machine (ZwickRoell, Ulm, Germany). Tendon biomechanical load-to-failure testing was performed at a rate of 0.1 mm/s, and axial force–displacement and stress–strain curves were plotted to obtain the parameters of the biomechanical properties using TestXpert II software (version 3.2, ZwickRoell, Ulm, Germany).

### 4.6. Statistical Analysis

Statistical analysis was performed using IBM SPSS Statistics version 26.0 (IBM, Armonk, NY, USA). Due to the small sample size, normality and homoscedasticity analyses of the data were not performed. As a result, nonparametric Kruskal–Wallis tests were performed directly to assess statistical significance. The post hoc Dunn’s test was performed for multiple comparisons, and the Bonferroni correction was applied to control for type I errors.

The multiple comparisons assessed the differences between the different groups (H, CP, and P) at each time point (3, 7, 14, 30, and 60 days post-injury) and between the different time points for each group for all variables. Differences were considered significant with an adjusted *p*-value less than 0.01. The data are presented as mean ± SD for all the studied variables.

## 5. Conclusions

In summary, this study presents a novel animal model of patellar tendinopathy induced by injecting a collagenase–Pluronic mixture into rats; this mimics the degeneration processes of tendinopathy and reproduces the main features of degenerative tendinopathy observed in human patients, such as tissue disorganization, significant changes in the expression of tissue structure components, neovascularization, acute inflammatory response, tensile strength variations, and the appearance of intra-tendinous calcifications. Additionally, using Pluronic hydrogel prevents collagenase leakage and damage to the surrounding tissues, which opens the door to the development of treatments targeted to specific and localized tissue areas. This novel in vivo model of collagenase–Pluronic hydrogel-induced patellar tendinopathy could provide insight into the pathogenesis of tendinopathy and could open up new avenues for developing innovative therapeutic strategies to improve tendon healing.

## Data Availability

The data are contained within the article or in the Supplementary Materials.

## Supplementary Materials

The following supporting information can be downloaded at https://www.mdpi.com/article/10.3390/ijms25031868/s1.
